# Analysis of social determinants of health and extreme climate events: Identifying vulnerable populations and health outcomes in Jacksonville

**DOI:** 10.1097/EE9.0000000000000410

**Published:** 2025-07-14

**Authors:** Yuanfang Ren, Esra Adiyeke, Ziyuan Guan, Yingbo Ma, Jiahang Yu, Christine Angelini, Tezcan Ozrazgat-Baslanti, Azra Bihorac

**Affiliations:** a Division of Nephrology, Hypertension, and Renal Transplantation, Department of Medicine, College of Medicine, University of Florida, Gainesville, Florida; b Intelligent Clinical Care Center, University of Florida, Gainesville, Florida; c Department of Environmental Engineering Sciences, University of Florida, Gainesville, Florida

## Abstract

**Background:**

Heatwaves and hurricanes pose risks to public health, especially for vulnerable populations.

**Methods:**

We assessed the impact of extreme weather events on health outcomes in socially vulnerable groups. We analyzed hospital mortality, acute stroke, myocardial infarction, and nonelective intensive care unit (ICU) admissions for 24,814 patients admitted to two hospitals in Jacksonville (2015–2017). We fitted modified Poisson regression models to assess differences relative to the heatwave and Hurricane Irma, presenting impacts with relative risks (RR) and 95% confidence intervals (CI).

**Results:**

There was no significant change in the rate of acute stroke or myocardial infarction during both the heatwave and Hurricane Irma. The rate of nonelective ICU admission showed no significant change during the heatwave and immediate posthurricane period (0–14 days after the hurricane) but increased during the extended posthurricane period (15–74 days after the hurricane) with an RR of 6.19 (95% CI = 1.25, 30.54) compared with the control period. Extreme climate events amplified disparities among vulnerable patient populations. During heatwave and extended posthurricane periods, the rate of acute stroke or myocardial infarction increased for elderly patients relative to younger patients with RRs of 1.43 (95% CI = 1.05, 1.94) and 1.53 (95% CI = 1.14, 2.07), and uninsured or Medicaid-insured patients relative to those with Medicare or private insurance with RRs of 1.71 (95% CI = 1.26, 2.33) and 1.88 (95% CI = 1.38, 2.59). Social vulnerability and air quality further impacted health outcomes during the extended posthurricane period, with a 0.1-unit increase in the Social Vulnerability Index score associated with a 65% decrease in risk of acute stroke or myocardial infarction with RR of 0.35 (95% CI = 0.16, 0.75) and a 69% decrease in risk of nonelective ICU admissions with RR of 0.31 (95% CI = 0.15-0.65), and a 1-unit increase in Air Quality Index value indicated a 2% increase in risk of acute stroke or myocardial infarction with RR of 1.02 (95% CI = 1.01, 1.03).

**Conclusions:**

Our findings suggest extreme climate events exacerbate negative health outcomes in socioeconomically vulnerable populations, underscoring the need for targeted public health interventions, improved healthcare access, and greater climate resilience in vulnerable communities.

**What this study adds**

While studies have established associations between extreme weather events and health outcomes, few examine how these effects are modulated by social determinants of health. We retrospectively studied Duval County-residing patients admitted at two University of Florida Health hospitals in Duval County. We analyzed extreme weather events impact on acute stroke, myocardial infarction, nonelective intensive care unit admission, and hospital mortality, focusing on differential effects based on age, race, insurance status, Social Vulnerability Index, and Air Quality Index. We showed that Hurricane Irma was associated with increases in nonelective intensive care unit admission rates and that both it and the heatwave exacerbated negative health outcomes in socioeconomically vulnerable populations.

## Introduction

Extreme weather events, such as hurricanes, wildfires, flooding, and heatwaves, have been influenced by climate change and have had a major economic impact in North America.^1^ In 2023, the United States (US) experienced 28 extreme weather and climate events, with a cost of approximately $93 billion according to a report published in 2024 by the National Centers for Environmental Information (NCEI).^2^ Beyond their economic toll, these events disrupt communities, strain health systems, and adversely affect public well-being through various pathways.

With escalating intensity, incidence, and duration, heatwaves stand out as a major climate-related hazard, posing substantial direct and indirect risks to human health.^3,4^ Extreme heat has become one of the leading weather-related causes of death in high-income countries, contributing to an estimated 1,373–1,992 deaths/year in the US.^5,6^ In addition to deteriorated mortality rates, extreme heat events were associated with increased emergency room and hospital visits, higher rates of cardiovascular and respiratory deaths, and elevated healthcare costs.^7^ Like heatwaves, hurricanes or tropical cyclones are also projected to intensify with climate change. Hurricanes are expected to increase in strength by 5%, and rainfall rates are projected to rise up to 15% around 2055, indicating a higher likelihood of future flooding.^8^ In US counties impacted by at least one tropical cyclone between 1988 and 2018, each additional day of cyclone exposure was associated with mildly elevated mortality rates in the following months due to conditions such as infectious, cardiovascular, and respiratory diseases.^9^

While extreme weather events affect all individuals within impacted areas, their consequences are not evenly distributed across communities and regions.^10,11^ Vulnerable populations, particularly those with social disadvantages, tend to experience disproportionately worse health outcomes from these environmental stressors.^12,13^ Despite growing recognition of these risks, significant gaps remain in the literature, particularly regarding differential impacts on socially vulnerable populations. While studies have established a broad association between extreme weather events and adverse health outcomes, few have focused on how these effects are modulated by social determinants of health, such as socioeconomic status, race, and access to healthcare. This study seeks to fill that gap by investigating how heatwaves and hurricanes have affected hospital mortality, acute stroke, myocardial infarction, and intensive care unit (ICU) admissions in Duval County, Florida, with a particular focus on socially vulnerable populations. Located on Florida’s Atlantic coast, Duval County is susceptible to hurricanes, storm surges, and frequent extreme heat events during summers.^14^ We analyzed health outcomes in an adult cohort residing in Duval County and hospitalized at two University of Florida Health (UFH) hospitals in Jacksonville, assessing how extreme weather events in 2015–2017 impacted different population groups over time.

## Methods

### Study area and climate events

**Figure 1.**
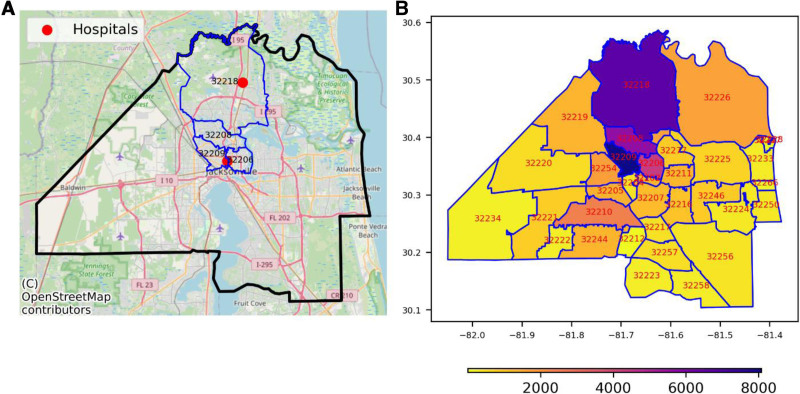
Study Area and Distribution of UF Health Hospital Admissions within Each Jacksonville Neighborhood. A, Map of Jacksonville Duval County. The black line indicates the boundary of the county. Red dots indicate UFH North and UFH JAX hospitals. The blue line highlights four neighborhoods. B, Distribution of number of hospital admissions. For (B), the x and y axes represent longitude and latitude, respectively. Higher numerical values (darker colors in the yellow-orange-purple scale) indicate a higher number of hospital admissions.

Duval County in Jacksonville encompasses a total area of approximately 2,378 km² and comprises 174 census tracts, with an estimated population of 912,000 in 2017. Within this area, there are two UFH hospitals: UFH Jacksonville and UFH North (Figure 1A). Four neighborhoods surrounding these hospitals—32218, 32208, 32209, and 32206—comprising 41 census tracts with an estimated population of 183,000 in 2017, were selected as the study area due to their proximity to the UFH hospitals and the higher concentration of UFH patients (Figure 1B).

We studied two types of extreme climate events, hurricanes and heatwaves, between 2015 and 2017. During this period, Jacksonville was impacted by two significant hurricanes: Hurricane Matthew and Hurricane Irma. Hurricane Matthew weakened to a Category 2 storm as it passed near Jacksonville on October 7, 2016, causing significant and concentrated damage in Jacksonville Beach.^15^ The downtown Jacksonville, near our study area, experienced maximum storm surges of three feet above normal tide level.^16^ Given that the primary affected area was Jacksonville Beach, where other hospitals are located, and that the flooding level in the study area was relatively minor, Hurricane Matthew was excluded from this study. Hurricane Irma made landfall on Marco Island as a Category 3 on 11 September 2017, and passed through Jacksonville on 11 September 2017, causing the worst flooding in the city’s 250-year history.^17^ The storm surges were about 4–6 feet above normal high tides in the St. Johns River, and flooding levels along the river in downtown Jacksonville had surpassed previous high records.^17,18^ Hurricane Irma caused 50 billion US dollars in damage in Florida,^19^ significantly higher than Hurricane Matthew’s 2.77 billion.^20^ As a result, Hurricane Irma was included in this study.

**Figure 2.**
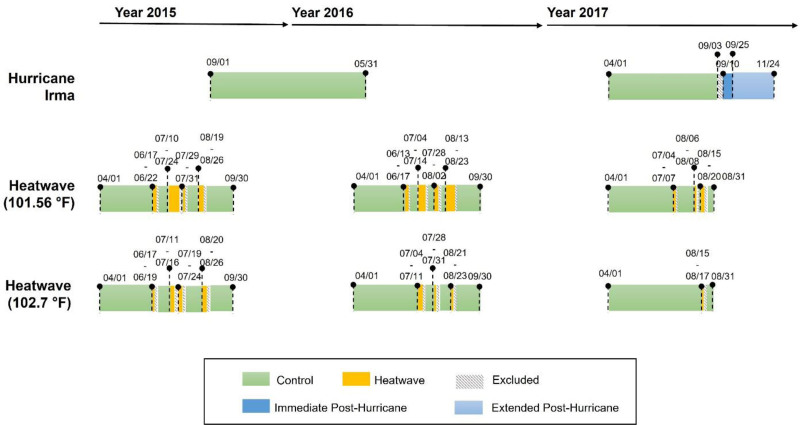
Timeframes of heatwave and hurricane periods. Heatwaves are identified using two different heat index thresholds (101.56 °F and 102.7 °F). For the heatwave event, only the start and end dates of analysis periods and heatwave periods are marked.

To assess the potential health impacts of Hurricane Irma, we defined the immediate and extended posthurricane periods and compared health outcomes with those during a control period in which the hurricane was not present and influencing the area. Due to the unavailability of stream gauge data necessary to accurately estimate the flood period days when water levels rose above normal, we adopted similar period lengths defined by Ramesh et al,^21^ who did have such data. They estimated the flood period to last 3 weeks starting from the landfall of Hurricane Harvey (category 4) and defined postflood periods as a 1-month postflood period and a second 1-month postflood period. Specifically, in this study, we defined the immediate posthurricane period as the day of hurricane landfall and the 14 days following its departure (September 10–September 25 of 2017), reducing the 3 weeks suggested by Ramesh et al^21^ to 2 weeks due to the difference in hurricane category. The extended posthurricane period was defined as the 60 days following the immediate posthurricane period (September 26–November 24 of 2017), thereby combining the two 1-month postflood periods described by Ramesh et al.^21^ One week before the hurricane landfall accounting for any preparatory measures and evacuations (i.e., washout period) was excluded from the analysis. Other periods, covering 1 September 2015, to 31 May 2016, and 1 April 2017, to 2 September 2017, were selected as control periods. A detailed breakdown of time periods is provided in Figure 2.

Located in the subtropics on Florida’s Atlantic coast, Jacksonville frequently experiences heatwaves during the summer. A heatwave is typically defined as a prolonged period of excessively hot weather. While there is no consistent definition of a heatwave, we used a similar method to Davis et al,^22^ who defined heatwaves as periods of at least 3 consecutive days in which the given threshold heat index is met or exceeded, with no more than one intervening day where the heat index falls below the threshold. The analysis was restricted to data from the months of April through September, while data in September 2017 was excluded due to the occurrence of Hurricane Irma. We selected two heat index thresholds based on the April–September data for our period of record: 101.56 °F, which is the 85th percentile, and 102.7 °F (90th percentile). We identified 11 heatwaves based on the 101.56 °F threshold, and 8 heatwaves based on the 102.7 °F threshold, accounting for 15% and 8% of the period of record, respectively (see Figure 2 and Table 1 for detailed heatwave time periods).

**Table 1.** Climate details for the 11 heatwaves using a 101.56 °F heat index (HI) threshold and the 8 heatwaves using a 102.7 °F HI threshold

Dates	Duration (days)	Mean HI	Max HI	Dates	Duration (Days)	Mean HI	Max HI
17–22 Jun 2015	6	104.5	107.6	17–19 Jun 2015	3	105.7	107.6
10–24 Jul 2015	15	104.2	109.2	11–16 Jul 2015	6	103.9	105.6
29–31 Jul 2015	3	102.5	104.9	19–24 Jul 2015	6	105.6	109.2
19–26 Aug 2015	8	103.3	105.8	20–26 Aug 2015	7	103.5	105.8
13–17 Jun 2016	5	103.0	106.3				
04–14 Jul 2016	11	103.3	106.3	04–11 Jul 2016	8	103.8	106.3
28 Jul–02 Aug 2016	6	103.8	106.3	28–31 Jul 2016	4	104.7	106.3
13–23 Aug 2016	11	102.1	104.4	21–23 Aug 2016	3	103.8	104.4
04–07 Jul 2017	4	103.0	104.4				
06–08 Aug 2017	3	102.2	102.7				
15–20 Aug 2017	6	103.0	103.8	15–17 Aug 2017	3	103.4	103.8

To assess the potential health impacts of heatwaves, we compared clinical outcomes during (and immediately after) heatwaves with those during control periods in which elevated heat and humidity were not present. The remaining days outside of heatwave time periods were assigned as the control periods. To reduce the potential lag impact of a heatwave, the 3 days following each heatwave were not assigned to control periods.

### Datasets

Using the UFH Integrated Data Repository as an honest broker for data de-identification, we created a longitudinal electronic health record (EHR) dataset for 47,013 hospital admissions from 22,170 adult (age ≥18 years) patients residing in Jacksonville, Duval County, who were admitted to the UFH between 2015 and 2017. Among the four selected neighborhoods, there were 24,814 hospital admissions from 10,770 patients during this period. The EHR dataset includes demographic information, vital signs, laboratory values, medications, diagnoses, and procedure codes for all index admissions, along with past medical history and follow-up data. The study was approved by the University of Florida Institutional Review Board and the University of Florida Privacy Office (IRB202200713) as an exempt study with a waiver of informed consent.

In addition to the EHR dataset, our study utilized an area-level social determinants of health dataset, specifically the social vulnerability index (SVI). SVI is a metric used to evaluate a community’s resilience in public health when facing external pressures, such as natural disasters, disease outbreaks, and other emergencies.^23^ SVI summary rankings were derived for each census tract based on 15 social variables, which can be grouped into four themes: socioeconomic status (SVI theme 1), household composition and disability (SVI theme 2), minority status and language (SVI theme 3), and housing type and transportation (SVI theme 4). SVI dataset included calculated theme rankings and overall tract rankings (SVI themes). A tool was developed to link the SVI dataset to EHR data, which geocodes the patients’ addresses into longitude and latitude coordinates, and then these coordinates are converted to census tracts, which are linked with SVI. The detailed workflow and data imputation process are outlined in Supplementary Figure 1; https://links.lww.com/EE/A361.

Environmental data, including weather data and Air Quality Index (AQI) data, were also incorporated into our analysis. Weather data were downloaded from the Visual Crossing website.^24^ The dataset, which was collected using six monitoring stations near Jacksonville, includes daily weather details such as temperature, heat index, precipitation, wind, etc. AQI data reporting outdoor air quality were obtained from the Environmental Protection Agency (EPA).^25^ AQI is calculated daily for each monitor for the criteria gases and PM_10_ and PM_2.5_ (both federal reference method and non-federal reference method ). The higher the AQI value, the greater the level of air pollution.

### Clinical outcomes

Primary outcomes were (1) acute stroke or myocardial infarction, identified with International Classification of Diseases, Ninth Revision, Clinical Modification (ICD-9-CM), or Tenth Revision (ICD-10) codes, acute stroke was defined by the presence of ICD-9 codes 430, 431, 432.x, 433.x1, 434 (excluding 434.x0), and 436, or ICD-10 codes I60.x, I61.x, I62.x, I63.x, and I64.x, myocardial infarction was defined using ICD-9 codes 410.x and 412.x, or ICD-10 codes I21.x, I22.x, and I25.2; (2) nonelective ICU admission, determined using admission priority, and date and time stamps for entry and exit to each station in hospital; and (3) hospital mortality, determined by the recorded date of death, obtained from hospital records and cross-referenced with the Social Security Death Index.

### Study design and statistical analysis

This study employed a before-and-after design to compare differences in clinical outcomes including acute stroke or myocardial infarction rate and nonelective ICU admission rate, before and after major climate events. Hospital mortality was not included due to its low prevalence and small sample size. This approach allowed us to evaluate changes in outcomes during and after climate events relative to baseline rates observed outside of the event period. To account for potential confounding factors, we applied a modified Poisson regression model incorporating sandwich error estimation^26^ using a generalized estimating equation. The model’s formula is as followed:


$$\begin{array}{l} \begin{array}{l} log_{e}\left[\pi_{i}\right]=\;\beta_{0}+\;\beta_{1}*Period+\;\beta_{2}*Sex+\;\beta_{3}*Race\;\\ +\;\beta_{4}*Elderly+\;\beta_{5}*CCI+\;\beta_{6}*Period*Sex\;\\ +\;\beta_{7}*Period*Race+\;\beta_{8}*Period*Elderly\;\\ \;+\beta_{9}*Period*\;Insurance_{Type}+\;\beta_{10}*Period*SVI_{Themes}\;\\ +\;\beta_{11}*Period*AQI_{prev\_7\_max}\;\\ +\;Seasonality+\;log_{e}\left(Daily\;total\;hospital\;admissions\right),\; \end{array} \end{array}$$

where $\pi_{i}$ indicates the probability of patient *i* experiencing the outcome (e.g., ICU admission), and the predictor variables are represented on the right side of the equation. The daily number of total hospital admissions is used as the offset, making the prediction represent the rate of the outcome, with the daily total hospital admissions as the denominator. The “Period” variable denotes the specific hurricane or heatwave periods (immediate postevent period, extended postevent period, and control period), with control period as the reference. The “sex” is categorized as female (reference category) or male. The “race” distinguishes between non-African American (reference category) and African American individuals. A numerical variable, “CCI” (Charlson Comorbidity Index),^27^ accounts for the patient’s prehealth condition. Interaction terms between the “period” and other variables are also included in the formula, allowing for a nuanced examination of whether the impact of climate events varies across different groups. The “insurance type” is categorized as uninsured/Medicaid and Medicare/Private (reference category) insurance. “SVI Themes” is a continuous variable ranging from 0 to 1, representing overall tract rankings. “AQI_prev_7_max_” is an integer value, representing the maximum AQI value within the past 7 days. Seasonality and weekly trends were modeled by incorporating years and months as categorical variables, along with a binary variable indicating whether the day was a weekend. The impact of climate events on health outcomes was assessed with relative risk (RR) with a 95% confidence interval (CI). The exponent of the coefficient $\beta_{1}$ in the equation provides the RR for the outcome (e.g., ICU admission rate) during each hurricane or heatwave period compared with the control period, while controlling for individual-level confounders and seasonality.

In addition to our primary analysis, we conducted sensitivity analyses to ensure robust findings. For Hurricane Irma, we defined the immediate hurricane period as the day of landfall and the 7 days following. Due to the immediate or short-lag health impacts of heat,^22,28,29^ we also examined the effects of a one-day lag for the heatwave event.

Census tracts were divided into three equal groups (tertiles) based on their SVI: low (SVI: 0.27–0.73), intermediate (0.73–0.89), and high (0.89–0.99). Patients were then grouped according to the SVI tertile of their residing census tracts and their clinical characteristics and outcomes were compared. The chi-square (*χ*²) test was used for comparison of categorical variables, while the Kruskal-Wallis test was employed for continuous variables. The threshold for statistical significance was set at a 2-tailed *P* value of 0.05. Analyses were performed using Python version 3.9 and R version 4.3.2.

## Results

### Clinical characteristics and outcomes of patients

Clinical characteristics and outcomes are presented in Table 2. Among 24,814 patient encounters in the UFH cohort, the mean age was 53 (standard deviation [SD], 17) years. Within this population, 14,308 (58%) encounters were female, 17,613 (71%) were African American, 6,688 (27%) were White, and 372 (1%) were Hispanic.

**Table 2.** Clinical characteristics and outcomes for two UF Health hospital admissions

Variables	All encounters (n = 24,814)	Encounters residing in low SVI tracts (n = 6596)^a^	Encounters residing in intermediate SVI tracts (n = 8787)^a^	Encounters residing in high SVI tracts (n = 9431)^a^	*P* value
Demographics					
Age, mean (SD), years	53 (17)	54 (17)	52 (17)	52 (17)	**<0.001**
Female sex, n (%)	14,308 (58)	3,688 (56)	5,136 (58)	5,484 (62)	**0.003**
Race, n (%)					
White	6,688 (27)	3,471 (53)	2,036 (23)	1,181 (13)	**<0.001**
African American	17,613 (71)	2,938 (45)	6,594 (75)	8,081 (85)	**<0.001**
Other	513 (2)	187 (3)	157 (2)	169 (2)	**<0.001**
Hispanic Ethnicity, n (%)	372 (1)	127 (2)	143 (2)	102 (1)	**<0.001**
Marital status, n (%)					
Married	5,161 (21)	2,203 (33)	1,789 (20)	1,169 (13)	**<0.001**
Divorced	8,605 (35)	2,208 (33)	3,026 (34)	3,371 (36)	**0.01**
Single	10,562 (43)	2,090 (32)	3,860 (44)	4,612 (49)	**<0.001**
Missing	486 (2)	95 (1)	112 (1)	279 (3)	**<0.001**
Primary english language, n (%)	24,581 (99)	6,521 (99)	8,745 (100)	9,315 (99)	**<0.001**
Insurance, n (%)					
Medicare	9,948 (40)	2,759 (42)	3,376 (38)	3,813 (40)	**<0.001**
Medicaid	11,881 (48)	2,662 (40)	4,374 (50)	4,845 (51)	**<0.001**
Private	2,624 (11)	1,074 (16)	917 (10)	633 (7)	**<0.001**
Uninsured	253 (1)	75 (1)	86 (1)	92 (1)	0.54
Not reported	108 (0.4)	26 (0.4)	34 (0.4)	48 (0.5)	0.38
Neighborhood					
Distance to facility, median (IQR), mile	8.2 (6.1, 9.3)	4.0 (2.5, 7.5)	7.6 (5.7, 8.7)	9.2 (8.6, 9.9)	**<0.001**
SVI theme 1, mean (SD)	0.81 (0.20)	0.56 (0.20)	0.85 (0.11)	0.95 (0.03)	**<0.001**
SVI theme 2, mean (SD)	0.77 (0.24)	0.51 (0.27)	0.80 (0.15)	0.92 (0.12)	**<0.001**
SVI theme 3, mean (SD)	0.55 (0.12)	0.49 (0.13)	0.60 (0.13)	0.56 (0.08)	**<0.001**
SVI theme 4, mean (SD)	0.69 (0.20)	0.58 (0.21)	0.61 (0.14)	0.84 (0.13)	**<0.001**
SVI themes, mean (SD)	0.80 (0.16)	0.57 (0.14)	0.81 (0.05)	0.94 (0.03)	**<0.001**
Comorbidities					
Charlson Comorbidity Index, median (IQR)	1 (0, 4)	1 (0, 4)	1 (0, 4)	1 (0, 4)	**<0.001**
Congestive Heart Failure, n (%)	4,480 (18)	1,144 (17)	1,514 (17)	1,822 (19)	**<0.001**
Diabetes, n (%)	6,789 (27)	1,723 (26)	2,374 (27)	2,692 (29)	**0.002**
Myocardial Infarction, n (%)	2,197 (9)	612 (9)	701 (8)	884 (9)	**0.001**
Hypertension, n (%)	5,716 (23)	1,354 (21)	1,949 (22)	2,413 (26)	**<0.001**
Liver disease, n (%)	3,056 (12)	791 (12)	1,077 (12)	1,188 (13)	0.51
Chronic kidney disease/end stage kidney disease, n (%)	8,793 (35)	2,064 (31)	3,149 (36)	3,580 (38)	**<0.001**
Outcomes					
Myocardial infarction or acute stroke, n (%)	3,417 (14)	914 (14)	1,104 (13)	1,399 (15)	**<0.001**
Non-elective admission to ICU, n (%)	3,465 (14)	999 (15)	1,209 (14)	1,257 (13)	**0.004**
Hospital mortality, n (%)	342 (1.4)	76 (1.2)	131 (1.5)	135 (1.4)	0.17

Bolded values indicate *P* value <0.05.

a Census tracts were divided into three equal groups (tertiles) based on their SVI, low (SVI: 0.27–0.73), intermediate (0.73–0.89), and high (0.89–0.99). Patients were then grouped according to the SVI tertile of their residing census tracts.

IQR indicates interquartile range.

Mean overall SVI scores were 0.57 (0.14) for low SVI tracts, 0.81 (0.05) for intermediate SVI tracts, and 0.94 (0.03) for high SVI tracts, where SVI ranges from 0 to 1 with higher values indicating higher social vulnerability. These three SVI groups exhibited distinct demographic characteristics. Patients residing in higher SVI census tracts tended to be younger (low: 54 vs. intermediate: 52 vs. high: 52 years), had a higher proportion of females (56% vs. 58% vs. 62%), a greater percentage of African Americans (45% vs. 75% vs. 85%), a higher prevalence of single-status families (32% vs. 44% vs. 49%), and were more likely to be covered by Medicaid (40% vs. 50% vs. 51%), all with a *P* value <0.05. Additionally, the median distance to the UFH facility was longer for patients in higher SVI tracts (low: 4.0 vs. intermediate: 7.6 vs. high: 9.2 miles, *P* < 0.001).

Patients residing in higher SVI tracts were more likely to have comorbidities such as congestive heart failure (low: 17% vs. intermediate: 17% vs. high: 19%), diabetes (26% vs. 27% vs. 29%), hypertension (21% vs. 22% vs. 26%), and chronic kidney disease or end-stage kidney disease (31% vs. 36% vs. 38%), all with a *P* value <0.05. While hospital mortality was similar in all groups (low: 1.2% vs. 1.5% vs. 1.4%), the nonelective ICU admission rate was slightly lower (15% vs. 14% vs. 13%, *P* < 0.001), and the incidence of myocardial infarction or acute stroke was slightly higher (14% vs. 13% vs. 15%, *P* = 0.004) in high SVI tract encounters.

**Figure 3.**
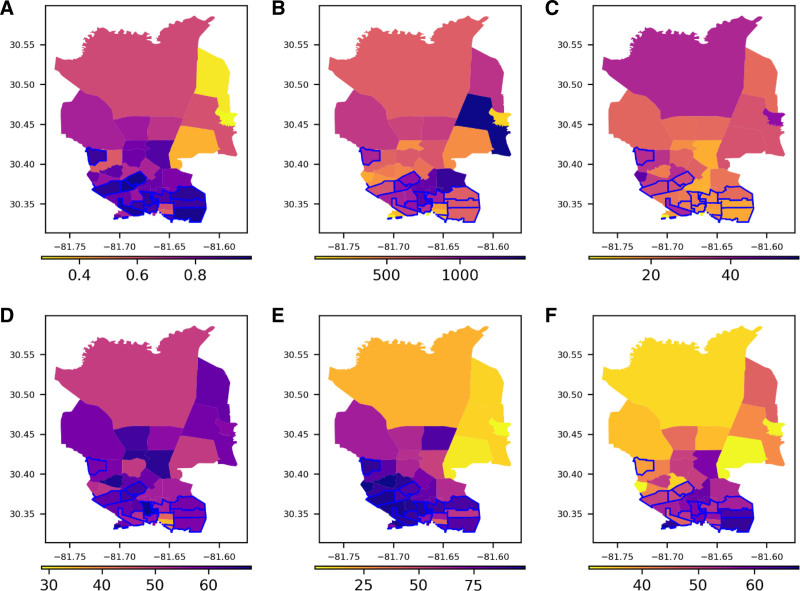
Distribution of clinical characteristics for UF Health Hospital admissions within each census district. A, Distribution of Social Vulnerability Index (SVI) ranging from 0 to 1. B, Number of hospital admissions. C, Percentage of patients ≥65 years of age. D, Percentage of female patients. E, Percentage of African American patients. F, Percentage of uninsured or Medicaid-insured patients. In panels (A) through (F), the blue line represents the top one-third of census districts with the highest SVI and numbers in x and y axes indicate longitude and latitude, respectively. Darker colors in the yellow-orange-purple scale represent higher values: higher social vulnerability in (A), a greater number of hospital admissions in (B), and higher percentages of elderly patients in (C), female patients in (D), African American patients in (E), and uninsured or Medicaid-insured patients in (F).

**Figure 4.**
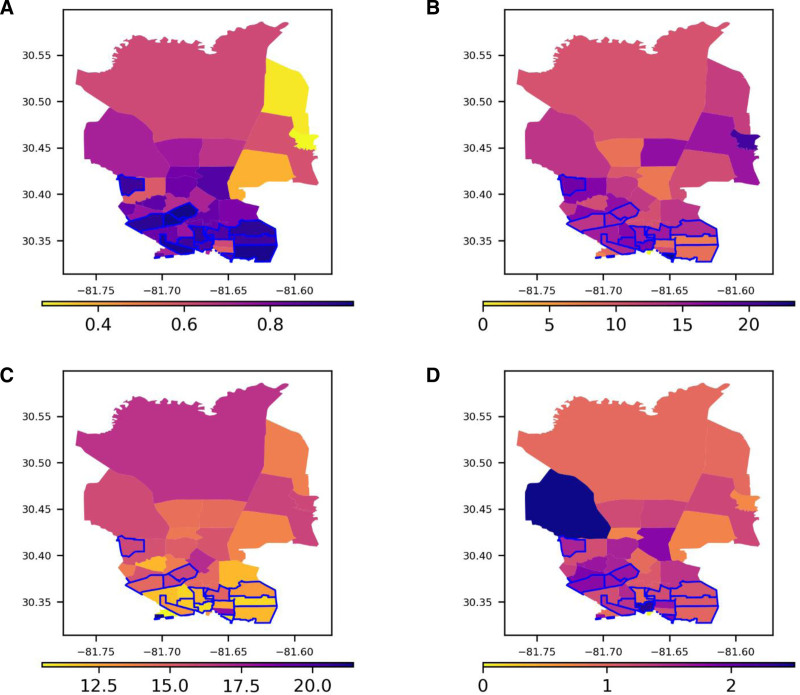
Distribution of clinical outcomes for UF Health Hospital admissions within each census district. A, Distribution of Social Vulnerability Index (SVI) ranging from 0 to 1. B, Percentage of acute stroke or myocardial Infarction. C, Percentage of nonelective intensive care unit (ICU) admission. D, Percentage of hospital mortality. In panels (A) through (F), the blue line represents the top one-third of census districts with the highest SVI and numbers in x and y axes indicate longitude and latitude, respectively. Darker colors in the yellow-orange-purple scale represent higher values: higher social vulnerability in (A), and higher percentages of patients with (B) acute stroke or myocardial Infarction, (C) nonelective ICU admission, and (D) hospital mortality.

The geographic distribution of patient clinical characteristics and outcomes is shown in Figures 3 and 4. A visual inspection of Figure 3A reveals that the top one-third of census districts with the highest SVI scores are primarily located in neighborhoods 32209 and 32206 and largely overlap with areas having a higher number of hospital admissions (Figure 3B). While the proportions of female and elderly patients were relatively homogenous, the distribution of African Americans and uninsured or Medicaid recipients varied significantly across census tracts, with higher concentrations in those with the highest SVI scores (Figure 3C–F). Nonelective ICU admission rates were generally lower in census tracts with higher SVI scores, whereas acute stroke or myocardial infarction and hospital mortality rates were generally higher (Figure 4).

### Impact of extreme climate events on health outcomes

**Figure 5.**
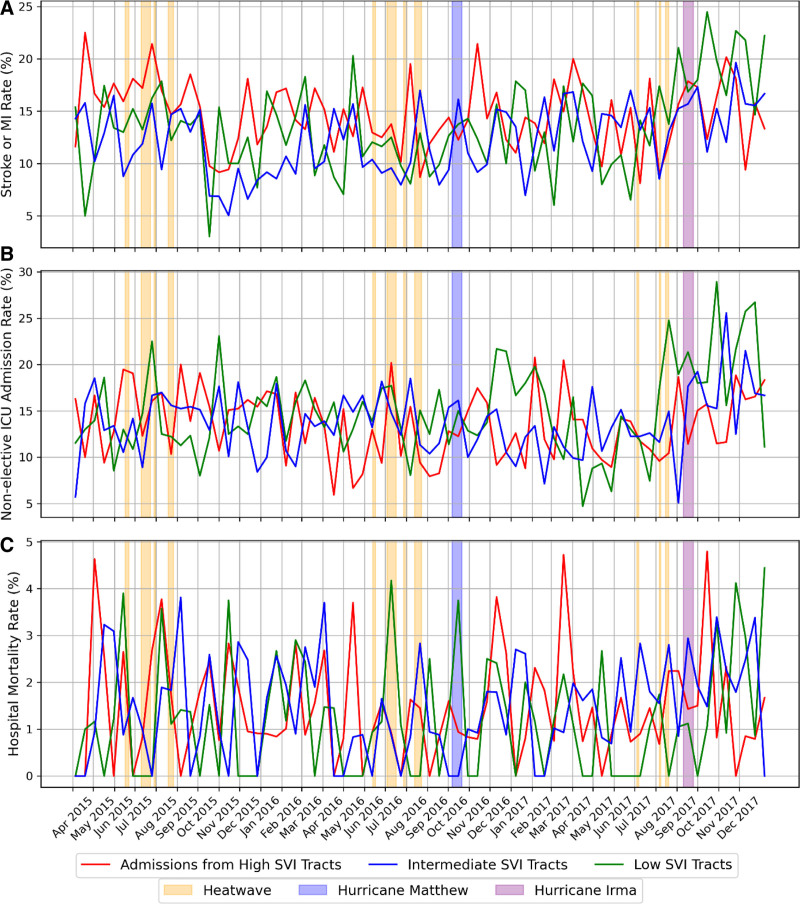
Distribution of bi-weekly clinical outcomes for UF Health Hospital admissions in low, intermediate, and high Social Vulnerability Index (SVI) Tracts from 2015 to 2017. A, Percentage of acute stroke or myocardial infarction. B, Percentage of nonelective intensive care unit (ICU) admissions. C, Percentage of hospital mortality. The heatwave period shown in the figure is determined using a heat index threshold of 101.56 °F. The hurricane period includes the day of hurricane landfall and the 14 days following its departure.

From April through September of 2015–2017, there were no obvious changes in all clinical outcomes during the heatwave period compared to nonheatwave periods across all patient groups (Figure 5). Although there were several peaks in hospital mortality rates during the heatwaves of 2015 and 2016, no consistent trend was observed. In the case of Hurricane Irma, the bi-weekly rates of acute stroke or myocardial infarction increased for patients from low SVI tracts after the hurricane (Figure 5A). Additionally, bi-weekly nonelective ICU admission rates rose for patients from low and intermediate SVI tracts following Hurricane Irma, but not for those from high-SVI tracts (Figure 5B). When compared to the six-month period before Hurricane Irma, hospital mortality rates generally increased during the extended posthurricane period, especially for patients from low and high SVI tracts, although this was not observed during the immediate posthurricane period (Figure 5C).

**Figure 6.**
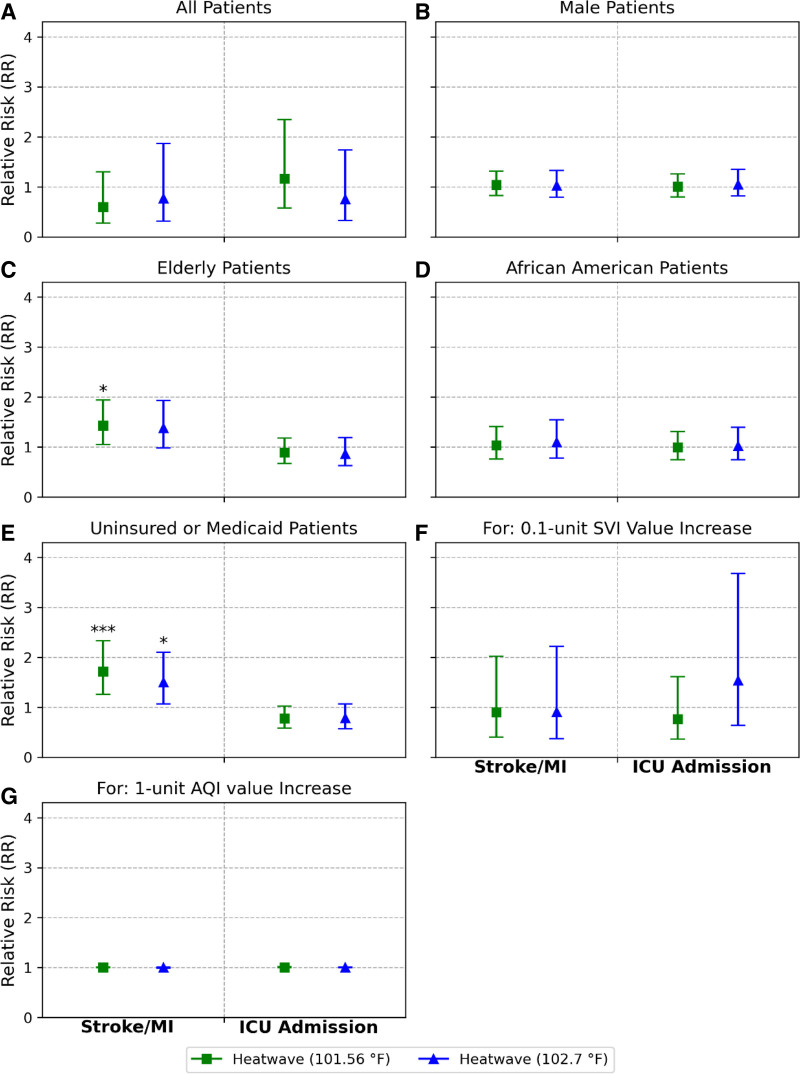
Relative risk with 95% confidence interval (CI) for clinical outcomes across vulnerable patient populations for heatwave. The relative risks were calculated for outcomes (acute stroke or myocardial infarction [MI] and Intensive Care Unit [ICU] admission) in 101.56 °F heatwave (square) and 102.7 °F heatwave (triangle) periods with respect to the control period, respectively. Panels (B–F) present the comparison results across various groups (B) male patients vs. female patients; (C) elderly patients vs. younger patients; (D) African American patients vs. non-African American patients; (E) uninsured or Medicaid insured patients vs. Medicare or privately insured patients; (F) 0.1-unit Social Vulnerability Index (SVI) value increase within each period; (G) 1-unit Air Quality Index (AQI) value increase within each period. The asterisk on top of the error bar represents a significant increase in relative risk compared to the control period.

**Figure 7.**
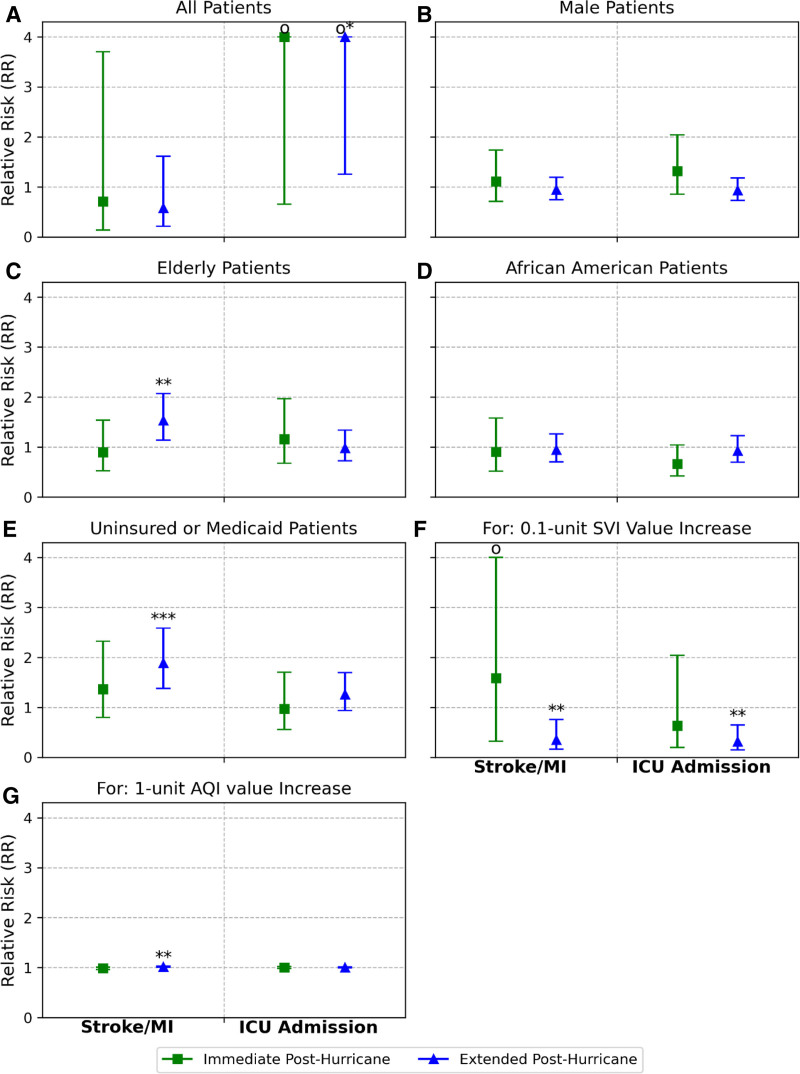
Relative risk with 95% confidence interval (CI) for clinical outcomes across vulnerable patient populations for Hurricane Irma. The relative risks were calculated for outcomes (acute stroke or myocardial infarction [MI] and Intensive Care Unit [ICU] admission) in immediate posthurricane (square) and extended post-hurricane (triangle) periods with respect to the control period, respectively. Panels (B–F) present the comparison results across various groups (B) male patients vs. female patients; (C) elderly patients vs. younger patients; (D) African American patients vs. non-African American patients; (E) uninsured or Medicaid insured patients vs. Medicare or privately insured patients; (F) 0.1-unit Social Vulnerability Index (SVI) value increase within each period; (G) 1-unit Air Quality Index (AQI) value increase within each period. The asterisk on top of the error bar represents a significant increase in relative risk compared to the control period. The circle above the error bar indicates a truncated upper bound relative risk score that is greater than 4.

Quantitative analyses of the impact of major extreme climate events, including heatwaves and Hurricane Irma, are presented in Figures 6 and 7. During the 101.56 °F heatwave, both the rates of acute stroke or myocardial infarction and nonelective ICU admissions showed no significant change compared with the control period, with RRs of 0.60 (95% CI = 0.27, 1.30) and 1.17 (95% CI = 0.58, 2.35), respectively (Figure 6A). Similarly, using a higher heat index threshold of 102.7 °F, the results remained consistent, with RR of 0.77 (95% CI = 0.32, 1.86) for the rate of acute stroke or myocardial infarction and RR of 0.76 (95% CI = 0.33, 1.73) for nonelective ICU admission rate (Figure 6A). For Hurricane Irma, the rate of acute stroke or myocardial infarction showed no significant change during the immediate posthurricane period and the extended posthurricane period compared with the control period, with RRs of 0.71 (95% CI = 0.14, 3.70) and 0.58 (95% CI = 0.21, 1.61), respectively. The rate of nonelective ICU admission showed no significant change during the immediate posthurricane period with an RR of 4.39 (95% CI = 0.65, 29.52) but increased during the extended posthurricane period with an RR of 6.19 (95% CI = 1.25, 30.54) compared with the control period.

Sensitivity analysis examining the effect of a one-day lag for the heatwave event and using a different hurricane period for Hurricane Irma provided consistent conclusions (Supplementary Figures 2 and 3; https://links.lww.com/EE/A361). Specifically, the rate of acute stroke or myocardial infarction showed no significant change during the 101.56 °F heatwave (RR = 0.79; 95% CI = 0.37, 1.72) and the 102.7 °F heatwave (RR = 0.78; 95% CI = 0.34, 1.79), as well as during the immediate posthurricane period (RR = 1.08; 95% CI = 0.11, 10.96) and the extended posthurricane period (RR = 0.44; 95% CI = 0.16, 1.24) compared with the control period. The nonelective ICU admission rate demonstrated no significant change during the 101.56 °F heatwave (RR = 1.22; 95% CI = 0.59, 2.55) and the 102.7 °F heatwave (RR = 0.79; 95% CI = 0.35, 1.81) and the immediate posthurricane period (RR = 4.21; 95% CI = 0.31, 56.85), but increased during the extended posthurricane period (RR = 5.62; 95% CI = 1.15, 27.56) compared with the control period.

### Influence on vulnerable patient populations

The impacts of major extreme climate events on specific patient subgroups are presented in Figures 6 and 7. Compared with the control period, elderly patients had a 43% higher risk of acute stroke or myocardial infarction than younger patients during the 101.56 °F heatwave, with RR of 1.43 (95% CI = 1.05, 1.94) (Figure 6C). However, no significant change was observed during the 102.7 °F heatwave. Similarly, the rate increased for uninsured or Medicaid-insured patients relative to those with Medicare or private insurance during both the 101.56 °F and 102.7 °F heatwaves, with RRs of 1.71 (95% CI = 1.26, 2.33) and 1.50 (95% CI = 1.07, 2.10) compared with the control period, respectively (Figure 6E). The rate of nonelective ICU admission showed no significant change among all patient subgroups (Figure 6B–G). Sensitivity analysis examining the effect of one-day lag for the heatwave event provided similar results (Supplementary Figure 2B–G; https://links.lww.com/EE/A361). Specifically, compared to the control period, the rate of acute stroke or myocardial infarction for elderly patients increased relative to younger patients during the 101.56 °F heatwave (RR = 1.52; 95% CI = 1.13, 2.05) and the 102.7 °F heatwave (RR = 1.53; 95% CI = 1.10, 2.13) (Supplementary Figure 2C; https://links.lww.com/EE/A361). Similarly, the rate increased for uninsured or Medicaid-insured patients with RRs of 1.57 (95% CI = 1.16, 2.14) and 1.55 (95% CI = 1.10, 2.16) during the two heatwaves, respectively (Supplementary Figure 2E; https://links.lww.com/EE/A361).

For Hurricane Irma, clinical outcomes showed no significant changes in the immediate posthurricane period among all patient subgroups compared with the control period (Figure 7B–G). However, Hurricane Irma was associated with an increase in adverse outcomes among specific patient groups in the extended posthurricane period. For example, compared with the control period, elderly patients had a 53% higher risk of acute stroke or myocardial infarction than younger patients, with an RR of 1.53 (95% CI = 1.14, 2.07) (Figure 7C). Similarly, uninsured or Medicaid-insured patients had an 89% higher risk relative to those with Medicare or private insurance with an RR of 1.88 (95% CI = 1.38, 2.59) (Figure 7E). Social vulnerability and air quality were also observed to impact the rate of acute stroke or myocardial infarction during the extended posthurricane period. Specifically, compared with the control period, a 0.1-unit increase in SVI score indicated a 65% decrease in risk with RR of 0.35 (95% CI = 0.16, 0.75), and a 1-unit increase in AQI value indicated a 2% increase in risk with RR of 1.02 (95% CI = 1.01, 1.03) during the extended posthurricane period (Figure 7F,G). The rate of nonelective ICU admission showed no significant change among all patient subgroups (Figure 7B–G). One exception is for social vulnerability, where a 0.1-unit increase in SVI score indicated a 69% decrease in risk with RR of 0.31 (95% CI = 0.15, 0.65) during the extended posthurricane period (Figure 7F), consistent with the observation shown in Figure 5B. Sensitivity analysis using a 7-day window to define the immediate posthurricane period provided consistent results (Supplementary Figure 3B-G; https://links.lww.com/EE/A361). Specifically, hurricane Irma amplified the disparity among elderly patients (RR = 1.41; 95% CI = 1.05, 1.90) and uninsured or Medicaid-insured patients (RR = 1.71; 95% CI = 1.25, 2.35) for the rate of acute stroke or myocardial infarction during the extended posthurricane period (Supplementary Figure 3C and 3E; https://links.lww.com/EE/A361). In addition, a 0.1-unit increase in SVI score indicated a 63% decrease in risk for the rate of acute stroke or myocardial infarction with RR of 0.37 (95% CI = 0.17, 0.82) and a 66% decrease in risk for the nonelective ICU admission with RR of 0.34 (95% CI: 0.16-0.70) during the extended post-hurricane period (Supplementary Figure 3F; https://links.lww.com/EE/A361). Furthermore, a 1-unit increase in AQI value indicated a 2% increase in risk with RR of 1.02 (95% CI = 1.01, 1.03) during the extended posthurricane period (Supplementary Figure 3G; https://links.lww.com/EE/A361).

## Discussion

In this analysis, we evaluated the patient clinical characteristics and outcomes using geospatial analysis and assessed the impact of extreme climate events on specific health outcomes (i.e., acute stroke, myocardial infarction, nonelective ICU admissions, and mortality). Our findings revealed significant geographic variation in these health outcomes closely linked to area-level social determinants of health. Our results highlighted that both heatwave and Hurricane Irma exacerbated negative health outcomes in socioeconomically vulnerable populations.

High SVI tracts had a higher concentration of African American patients and uninsured or Medicaid-insured patients. Patients from high SVI tracts were more likely to have chronic conditions, exhibit a higher number of hospital admissions, and experience acute stroke or myocardial infarction or in-hospital mortality. These findings suggest that areas of greater social vulnerability are associated with higher healthcare utilization and worse patient outcomes, possibly due to the higher prevalence of chronic conditions and reduced access to preventive care. Numerous studies have demonstrated that populations in high SVI areas tend to have poorer health outcomes, such as higher rates of hospital admissions, ICU stays, and mortality.^30–32^ For example, Kind et al^30^ highlighted that individuals residing in socially vulnerable neighborhoods are at greater risk for hospital readmissions and poor postacute care outcomes. Grunwell et al^31^ identified neighborhood hot spots for pediatric ICU admissions for asthma, which were strongly associated with higher SVI scores.^31^

Previous studies have demonstrated that heatwaves are associated with significant increases in daily hospital admissions, emergency admissions, and admissions for ischemic stroke and heat stroke.^33–35^ However, we did not find an association between heatwave and acute stroke or myocardial infarction in our study. One potential reason for this discrepancy may be that our data on hospital admissions did not capture cases treated exclusively in the emergency room. Few studies have investigated the association between heatwaves and ICU admissions. For instance, Troung et al^36^ observed that heat waves were associated with a lower rate of daily ICU admissions, compared with cold waves. However, our study did not find an association between heatwave and nonelective ICU admissions. One possible explanation might be that our selected control period still had warm temperatures rather than cold wave conditions, which may have masked the differences in health outcomes.

Exposure to hurricanes has been shown to be associated with increased risk of emergency visits,^21^ ischemic strokes,^37^ and ICU admissions.^38^ In this study, we did not find a significant change in the rate of acute stroke or myocardial infarction during and after the hurricane but observed an increase in the nonelective ICU admissions during the extended posthurricane period. Nevertheless, the wide CI of RR indicates the result should be considered with caution. Interestingly, higher SVI and higher social vulnerability were associated with a decrease in the rates of acute stroke or myocardial infarction and nonelective ICU admissions during the extended posthurricane period. This observation appears inconsistent with much of the existing literature, which generally asserts that higher social vulnerability correlates with worse health outcomes during and after extreme weather events.^39–41^ However, Ramesh et al^42^ identified that persons from the least vulnerable census tracts were more likely to visit an emergency department compared with those from high SVI tracts, with this trend primarily driven by themes related to socioeconomic status, housing type, and transportation. This discrepancy highlights the complexity of healthcare-seeking behaviors and suggests that social determinants can significantly influence health outcomes and access to care following extreme weather events. Additionally, psychological stress induced by hurricanes and their aftermath should be considered as a significant mechanism influencing health outcomes. Psychological stress can exacerbate preexisting health conditions and trigger acute cardiovascular events. The immediate stress of the disaster could lead to an initial surge in healthcare visits, while prolonged stress and resource depletion might contribute to adverse health effects over time. High SVI populations may experience intensified psychological stress due to greater exposure to the hardships caused by hurricanes, which could impact their health-seeking behaviors and access to care. Addressing psychological stress and its effects is crucial in future studies for understanding the full impact of extreme weather events on vulnerable populations.

Extreme climate events amplified disparities among vulnerable patient populations, particularly elderly individuals and uninsured or Medicaid-insured patients. These disparities were particularly pronounced during the heatwave period and the extended posthurricane period, with elderly patients experiencing a marked rise in acute stroke or myocardial infarction events compared with younger patients. This is consistent with literature demonstrating that elderly populations, males, and those with lower socioeconomic status are at greater risk of adverse outcomes during climate events.^39–41,43^ For example, He et al^39^ observed a significant increase in stroke risk on days with extreme nighttime heat, particularly among older individuals, females, and patients with mild stroke symptoms. A study by Gao et al^43^. found that recurrent tropical cyclones and extreme heat exacerbated mortality from heart diseases and strokes among older populations, with co-occurring hurricanes and extreme heat having an even more severe impact.^43^ Similarly, the significant rise in acute stroke or myocardial infarction rates among uninsured or Medicaid-insured patients underscores the connection between economic vulnerability and health outcomes during climate events. Research has shown that lower-income populations often have limited access to healthcare, making them more susceptible to the adverse effects of environmental stressors.^44–46^

Hurricanes and associated floodwaters have been reported to stir up pollutants, release toxic substances, and cause long-lasting effects on air quality due to the damage to homes, buildings, and infrastructure. Our study observed that air pollution during extended posthurricane periods is associated with an increased risk for acute stroke or myocardial infarction. The result is consistent with previous studies that have established cardiovascular disease as a significant consequence of air pollution.^47–49^ Both short-term and long-term exposure to air pollution have been linked to various cardiovascular conditions, such as heart attacks, heart failure, high blood pressure, and strokes.^47,48^

Our findings align with the current scholarship and support the need for policy interventions to improve healthcare access for these vulnerable groups, particularly in the context of climate resilience. A community can focus on strengthening social support networks, building climate-resilient infrastructure, and establishing community-based services that provide accessible evacuation and emergency response. Creating more green spaces can improve air quality and mitigate the urban heat island effect. Implementing emergency preparedness programs and adopting data-driven approaches that incorporate medical data, SVI, and environmental monitoring systems that can better predict, issue early warning, and respond to the health needs of vulnerable populations, thus helping to tailor interventions more effectively. Additionally, it is crucial to conduct targeted public health campaigns to raise awareness about the risks associated with extreme weather events and the importance of seeking medical care promptly.

Our study has several limitations. First, while we focused on neighborhoods close to UFH hospitals, Duval County contains other hospitals, which may limit our ability to precisely determine population rates of hospitalization based solely on UFH data. Additionally, focusing on a small neighborhood results in a smaller sample size, which limits the generalizability of our findings. Second, we focused on a limited set of health outcomes related to extreme climate events. Previous research has demonstrated associations between hurricanes and a wider range of health issues, including asthma, diarrheal diseases, pregnancy complications, acute respiratory infections, dehydration, heat-related illnesses, and mental health.^21^ Future studies should explore the impacts of hurricanes and heatwaves on these broader health outcomes. Third, we combined the hurricane and immediate posthurricane periods due to limited data on the precise ending times of hurricanes across different census tracts. Some studies have utilized satellite data to assess population exposure to flooding and US Geological Survey real-time stream gauge data to determine exposure periods more accurately.^21^ Incorporating such methods in future analyses could improve precision. Lastly, many hurricane- and heatwave-related illnesses are acute conditions, and focusing solely on hospital admissions may overlook cases treated in emergency departments. Future research should consider emergency admissions to capture the full spectrum of climate-related health impacts.

## Conclusions

Our study highlights the substantial impact of extreme climate events on health outcomes in socioeconomically vulnerable populations. High SVI tracts, with higher concentrations of African American and uninsured or Medicaid-insured patients, experienced more chronic conditions, increased hospital admissions, and worse health outcomes. Climate events further amplified these disparities. These findings underscore the need for targeted public health interventions, improved healthcare access, and greater climate resilience in vulnerable communities. Future research should explore a broader range of climate-related health outcomes and employ more precise methods to assess population exposure, using larger cohorts and incorporating both hospital and emergency department admissions to improve the generalizability and robustness of the findings.

## Conflicts of interest statement

The authors declare that they have no conflicts of interest with regard to the content of this report.

## ACKNOWLEDGMENTS

*We would like to acknowledge the Intelligent Clinical Care Center research group for the support provided for this study. We acknowledge the University of Florida Integrated Data Repository (IDR), the UF Health Office of the Chief Data Officer for providing the UFH analytic dataset.*

## Supplementary Material


