# Supplementary material for: Analysis of social determinants of health and extreme climate events: Identifying vulnerable populations and health outcomes in Jacksonville
Source: Environ Epidemiol. 2025 Jul 14;9(4):e410. doi: 10.1097/EE9.0000000000000410 (PMC12263012; doi:10.1097/EE9.0000000000000410)
Supplement: Supplementary file 1 [file ee9-9-e410-s001.docx]

**Supplement**

Yuanfang Ren, Esra Adiyeke, Ziyuan Guan, Yingbo Ma, Jiahang Yu, Christine Angelini, Tezcan Ozrazgat-Baslanti, Azra Bihorac.  **Analysis of social determinants of health and extreme climate events.**

This supplemental material has been provided by the authors to give readers additional information about their work.


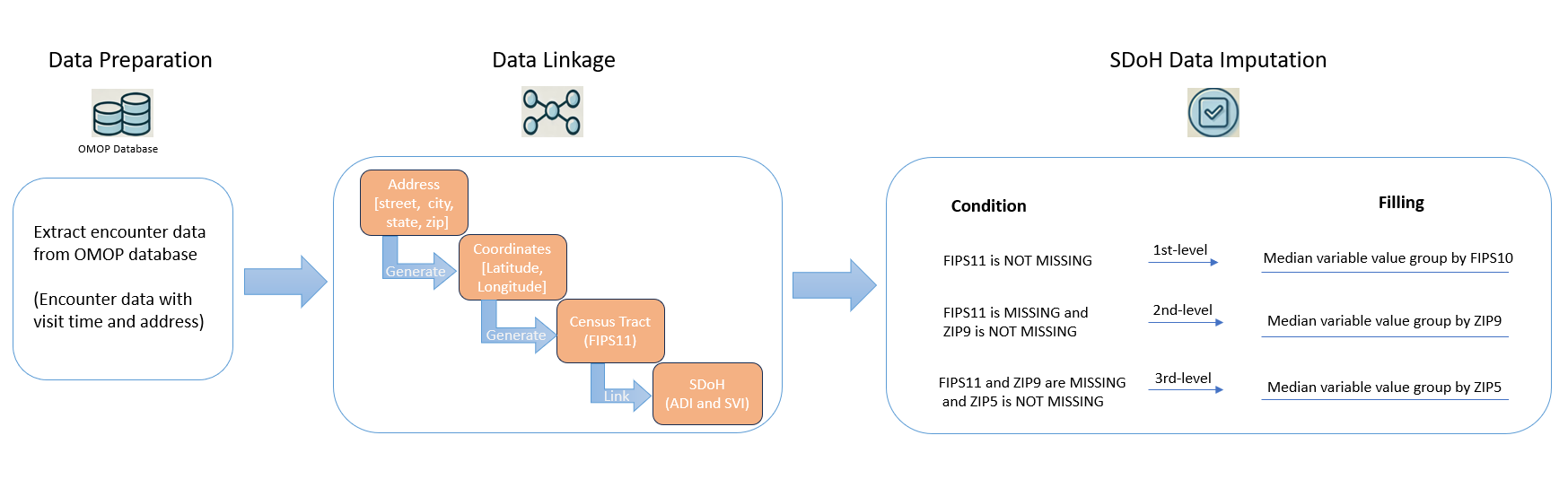


**Supplementary Figure 1**. Data Preparation and Processing Flow Chart


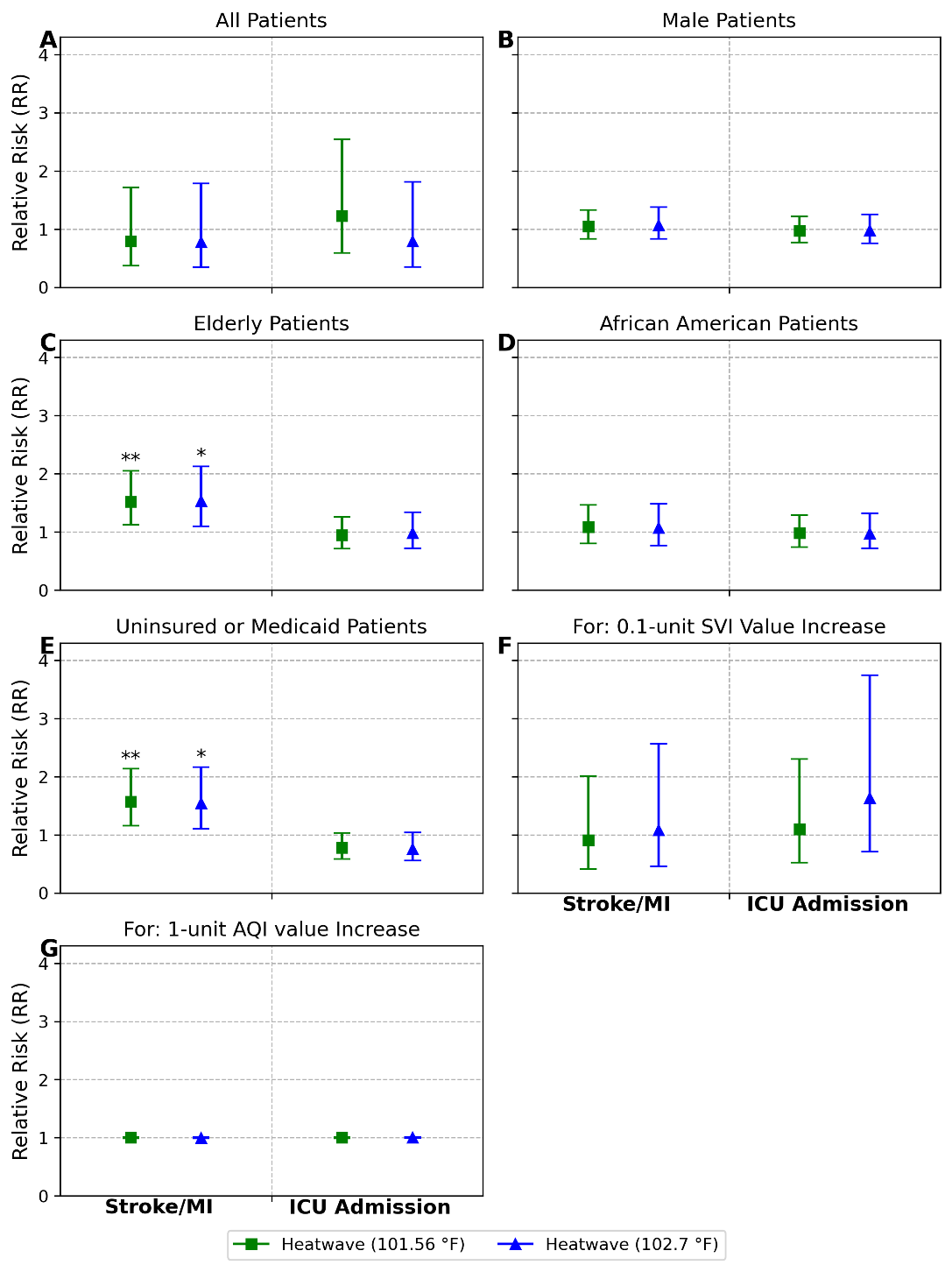


**Supplementary Figure 2.** Relative Risk with 95% Confidence Interval (CI) for Clinical Outcomes across Vulnerable Patient Populations for Heatwave in Sensitivity Analysis by Using a One-day Lag. The relative risks were calculated for outcomes (acute stroke or myocardial infarction [MI] and Intensive Care Unit [ICU] admission) in 101.56 °F heatwave (square) and 102.7 °F heatwave (triangle) periods with respect to control period, respectively. Panels (B-F) present the comparison results across various groups (B) male patients vs. female patients; (C) elderly patients vs. younger patients; (D) African American patients vs. non-African American patients; (E) uninsured or Medicaid insured patients vs. Medicare or privately insured patients; (F) 0.1-unit Social Vulnerability Index (SVI) value increase within each period; (G) 1-unit Air Quality Index (AQI) value increase within each period. Asterisk on top of the error bar represents a significant increase in relative risk compared to the control period. The circle above the error bar indicates a truncated upper bound relative risk score that is greater than 4.


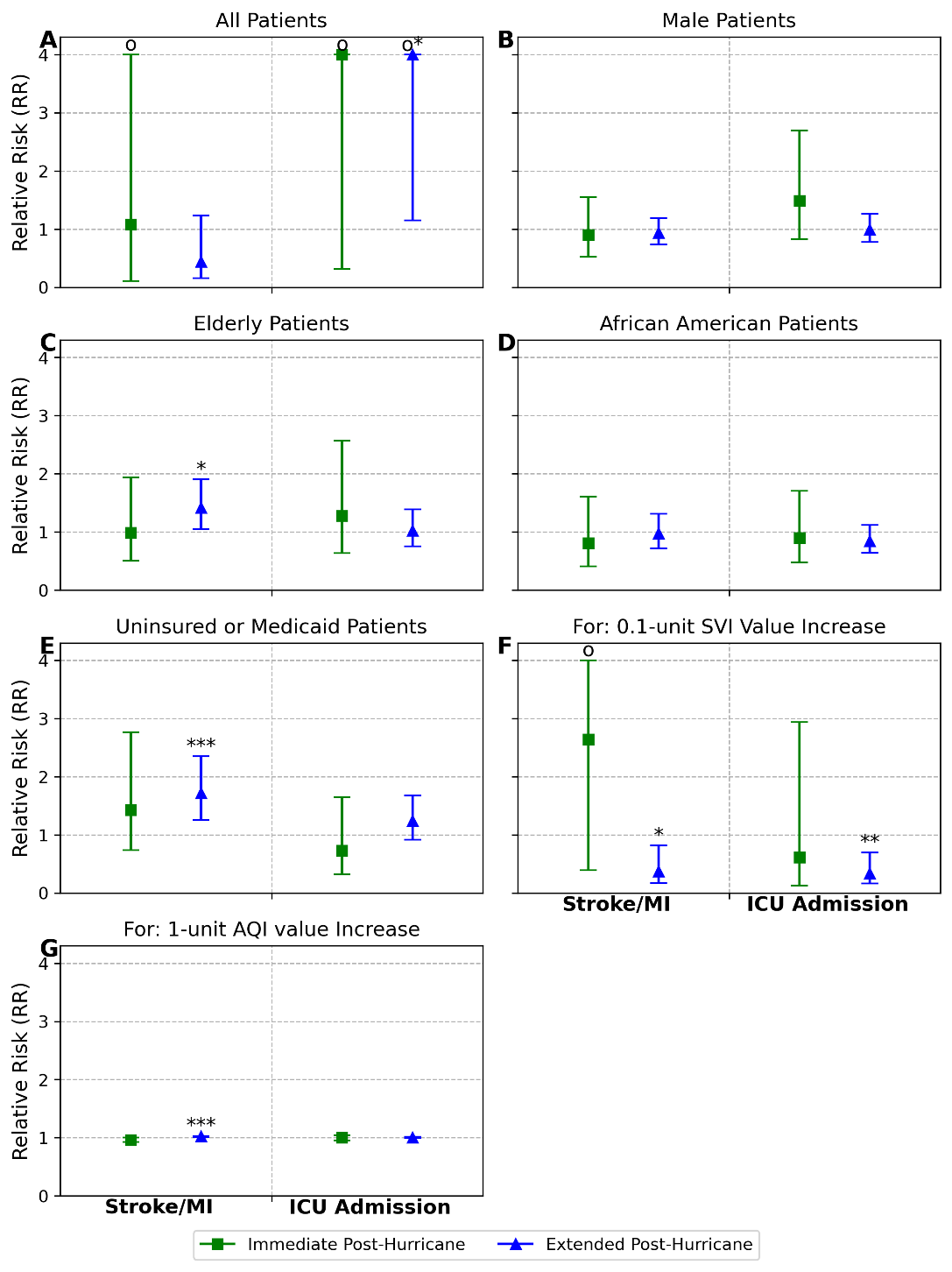


**Supplementary Figure 3.** Relative Risk with 95% Confidence Interval (CI) for Clinical Outcomes across Vulnerable Patient Populations for Hurricane Irma in Sensitivity Analysis by Using a 7-day Window for Immediate Post-hurricane Period. The relative risks were calculated for outcomes (acute stroke or myocardial infarction [MI] and Intensive Care Unit [ICU] admission) in immediate post-hurricane (square) and extended post-hurricane (triangle) periods with respect to control period, respectively. Panels (B-F) present the comparison results across various groups (B) male patients vs. female patients; (C) elderly patients vs. younger patients; (D) African American patients vs. non-African American patients; (E) uninsured or Medicaid insured patients vs. Medicare or privately insured patients; (F) 0.1-unit Social Vulnerability Index (SVI) value increase within each period; (G) 1-unit Air Quality Index (AQI) value increase within each period. Asterisk on top of the error bar represents a significant increase in relative risk compared to the control period. The circle above the error bar indicates a truncated upper bound relative risk score that is greater than 4.
